# Keratinolytic protease from *Pseudomonas aeruginosa* for leather skin processing

**DOI:** 10.1186/s43141-021-00149-8

**Published:** 2021-04-06

**Authors:** Yeasmin Akter Moonnee, Md Javed Foysal, Abu Hashem, Md Faruque Miah

**Affiliations:** 1 Department of Genetic Engineering & Biotechnology, Shahjalal University of Science & Technology, Sylhet, 3114 Bangladesh; 2 School of Molecular and Life Sciences, Curtin University, Perth, WA 6102 Australia; 3 Microbial Biotechnology Division, National Institute of Biotechnology (NIB), Savar, Dhaka, 1349 Bangladesh

**Keywords:** Bacteria, Biodegradation, Fermentation, Leather industry, Keratin waste

## Abstract

**Background:**

The leather industry generates huge volume of waste each year. Keratin is the principal constituents of this waste that is resistant to degradation. Some bacteria have the ability to degrade keratin through synthesis of a protease called keratinase that can be used as sources of animal feed and industrial production of biodiesel, biofertilizer, and bioplastic. Majority of the studies focused on keratin degradation using gram-positive bacteria. Not much of studies are currently available on production of keratinase from gram-negative bacteria and selection of best parameters for the maximum production of enzyme. The aim of this study was to isolate and characterize both groups of bacteria from soil for keratinase and optimize the production parameters.

**Results:**

A total of 50 isolates were used for initial screening of enzyme production in skim milk, casein, and feather meal agar. Out of 50, five isolates showed significantly higher enzyme production in preliminary screening assays. Morphological and biochemical characterization revealed 60% of the isolates as gram-negative bacteria including two highest enzyme-producing isolates. The isolates were identified as *Pseudomonas aeruginosa* through sequencing of 16S rRNA gene. Maximum production of enzyme from *P. aeruginosa* YK17 was achieved with 2% chicken feather, beef extract, and ammonium nitrate as organic and inorganic nitrogen sources and glucose as a carbon source. Further analysis revealed that 3% inoculum, 40 °C growth temperature and 72-h incubation, resulted in maximum production of keratinase.

**Conclusion:**

The overall results showed significant higher production of enzyme by the *P. aeruginosa* YK17 that can be used for the degradation of recalcitrant keratin waste and chemical dehairing in leather industries, thereby preventing environmental pollution.

**Supplementary Information:**

The online version contains supplementary material available at 10.1186/s43141-021-00149-8.

## Background

In Bangladesh, the leather industry accounts for 3.5% of the annual exports, employs over 850,000 workers, and occupies 3.0% of the world total leather market [1]. Poultry is another fastest growing industry in Bangladesh, accounting for 14% of total livestock outputs and 37% of total meat production [2]. Each year, 60,000 t of skins and raw hides are processed in the leather industry by dehairing chemicals resulting in release of 95,000 L of untreated effluents in rivers and open environments daily. A further 15,600 t of poultry wastes also pose constant threat to the environment and ecosystem [3]. These effluents spread heavy metals and contaminants to other water bodies, agricultural crops, and aquatic animals as well as into humans and cause serious health problems [4–6].

A diverse type of wastes is produced in leather and poultry industry during the process of hides and skins. This waste contain over 90% of keratin that is highly inert, water insoluble, and non-biodegradable by most proteolytic enzymes such as trypsin, pepsin, and papain [7, 8]. The traditional methods for the disposal of keratin including incineration, land filling, and chemical treatments that are expensive, non-sustainable, and energy intensive result in loss of natural resources and environmental pollution [8–10]. The enzyme that performs synthetic and biodegradative functions named as protease is found ubiquitously in plants, animals, and microbes [11]. Protease is one of the three most industrially important enzymes, which accounts for 20% of the global retail market with value of USD 3 billion [11, 12]. Proteases have the ability to control the activation, synthesis, and turnover of proteins to regulate physiological processes including birth, aging, and death [12], as well as play a vital role in disease control and drug discovery [13, 14]. However, numerous disulfide bonds make keratin insoluble and resistant to protease degradation [15]. Keratinolytic proteases mostly belong to serine and metalloprotease with broad substrate specificity that have the ability to hydrolyze both soluble (casein, gelatin, albumin) and insoluble (feather, silk, wool) proteins [16].

Among the various sources, microbial keratinolytic proteases are considered as a potential alternative for chemical approaches for the biodegradation of keratin due to their availability, rapid growth, high yield, less space requirement, cost, and sustainability [17, 18]. Microbes produce two-thirds of commercial proteases globally [19]. Bacteria mainly from the environment can synthesize keratinolytic proteases in bulk amount. Among them, gram-positive *Actinomycetes*, *Streptomyces*, *Arthrobacter*, *Bacillus*, and *Clostridium* are the most active and dynamic keratinolytic protease producers that hydrolyse insoluble keratin into simple molecules with high substrate specificity and catalytic activity [14, 20, 21]. Some reports are also available on keratin degradation using gram-negative bacteria [22], however resulted in low production of enzyme. On this backdrop, the present study aimed to isolate and characterize both gram-negative and gram-positive keratinolytic protease bacteria to selectively identify the best enzyme-producing isolate and select the best parameters among several tested combinations for the optimum production of enzyme for the leather industry.

## Methods

### Collection of samples

The soil samples were collected from waste disposal sites of Hazaribagh tannery (23.7361 N, 90.3631 E), Dhaka, in the month of September, 2018. A total of six samples were collected from two different waste disposal sites, three from each site. Collected samples were taken in a sterile ziplock bag and transported directly to the laboratory.

### Isolation of bacteria

Isolation of soil bacteria was performed by spread plate method. One gram of soil from each of the six samples was poured into 9 ml of sterile distilled water and homogenized by vortexing. Serial dilutions (10^−1^–10^−5^) was performed using deionized distil water, and 100 μl of diluted samples was plated in nutrient agar using an L-shaped glass rod. The plates were incubated overnight at 37 °C followed by picking of single colony and sub-culturing on nutrient agar.

### Screening for the keratinase-producing bacteria

#### Screening on skim milk agar

Skim milk agar (SMA) (Himedia, India) medium was used for the screening of keratinolytic bacteria [20]. A total of 50 single colonies were cultured in SMA and incubated at 37 °C for 48 h. Trichloroacetic acid (10%) was poured in the media to visualize the zone of clearance. The bacteria that showed a clear zone in the plate were primarily considered as positive for keratinolytic activity.

#### Screening on casein agar

Casein agar medium (Himedia, India) was used for screening keratinolytic bacteria according to the method described earlier [23]. A total of 50 single colonies were streaked on the casein agar plate and incubated at 37 °C for 48 h. Proteolytic activity was confirmed by a zone of clearance in the agar plate.

#### Screening on feather meal agar

The feather meal was prepared from native chicken feathers as described earlier [24]. Briefly, finely pieced feather was defatted in chloroform to methanol (1:1 v/v) for 48 h, followed by chloroform to acetone to methanol (4:1:3 v/v/v) for another 48 h, and then rinsed in sterile water, dried for 24 h at 37 °C, and ground in a mortar-pestle to obtain a powdered feather meal. This agar medium was used for the confirmation of keratinase-producing bacteria. The composition and concentration of feather agar plate was maintained according to the method described earlier (1.0% feather meal, 0.05% of NH_4_Cl and NaCl, 0.04% K_2_HPO_4_, 0.03% KH_2_PO_4_, 0.01% of MgCl_2_, 0.01% yeast extract, pH 7.5) [25]. A total of 16 single bacterial colonies were transferred to a feather meal agar after checking the activity on skim milk agar medium and casein agar. The culture plates were was incubated at 37 °C for 48 h.

### Identification of potent keratinolytic bacterial isolates

The identification of prospective keratinolytic bacteria was carried out by morphological and biochemical tests according to Bergey’s Manual Systematic Bacteriology [26] as well as 16S rRNA amplification using universal primers.

#### Biochemical tests

The primary identification of 50 bacterial colonies was performed based on Gram’s test, Gram staining, catalase, oxidase, indole production, nitrate reduction, gelatin and starch hydrolysis, salt tolerance, citrate utilization, and growth on MacConkey agar.

#### DNA extraction and PCR amplification

Bacterial DNA from overnight culture was extracted using phenol-chloroform isoamyl alcohol (25:24:1) [27]. Extracted DNA was stored at – 20 °C until further use. PCR amplification of bacterial 16S rRNA was carried out with 27F (5′-AGA GTT TGA TCC TGG CTG AG-3′) and 1492R (5′-GGC TAC CTT GTT ACG ACT T-3′) universal primers. PCR master mix was prepared as 25 μl final volume with 12.5 μl 2X master mix (Thermo Fisher Scientific, USA), 1 μl of each forward and reverse primers, 2 μl of template DNA, and 8.5 μl of nuclease-free water. A total of 40 cycles of amplification reactions was carried out in an ASTEC thermal cycler (Gene Atlas, Japan). The amplification conditions was maintained as follows: initial denaturation at 95 °C for 30 s, denaturation 95 °C for 1 min, annealing at 56 °C for 30 s, extension at 72 °C for 1.5 min, and a final extension at 72 °C for 10 min. PCR products were separated in 1.5% agarose gel with 1-kb DNA ladder (Thermo Fisher Scientific, USA) and visualized under gel documentation system.

#### Sequence analysis

The initial quality of Sanger sequence was checked using FastQC pipeline. Editing and removing of low-quality bases and de-novo assembly was performed using Geneious (vR11.1) bioinformatics software. ClustalW Multiple sequence alignment (MSA) and neighbor-joining phylogenetic tree contraction were performed in MEGA 7.0 with 1000 bootstrap replications [28].

### Enzyme assay

#### Inoculum preparation and fermentation

Pure cultures of eight identified potent keratinolytic bacteria were used as inoculant for shake flask fermentation of keratinase where each sample was run in triplicate. First, bacterial inoculum was prepared in nutrient broth culture by incubating overnight at 37 °C in a rotary shaking incubator. Submerged fermentation was carried out in an Erlenmeyer shake flask fermenter. Basal modified medium was used for keratinase production containing feather meal powder (10 g/l), NH_4_Cl (1.0 g/l), NaCl (1.0 g/l), KH_2_PO_4_ (0.8 g/l), K_2_HPO_4_ (0.6 g/l), MgCl_2_.6H_2_O (0.5 g/l), yeast extract (0.2 g/l), and pH (7.5) with 5% bacterial inoculum [29]. The fermented organisms were cultivated in 250-ml cotton-plugged Erlenmeyer flasks after 72 h shaking at 150 rpm. After incubation, fermented broth was centrifuged at 8000 rpm for 15 min at 4 °C. Cell-free supernatant was collected and preserved for the estimation of keratinase activity as described previously [30].

#### Assay for keratinase activity

Keratinase activity was assayed with keratin azure (Sigma Aldrich, Germany) as a substrate [31]. Briefly, the reaction mixture containing 1.0 ml enzyme preparation was transferred into 1.0 ml 50 mM Tris-HCl buffer suspended into 5 g keratin azure powder (pH 8.0). The mixture was incubated for 30 min at 50 °C, and the reaction was then stopped by the addition of 2 ml of 10% (w/v) trichloroacetic acid (TCA). After centrifugation at 1500 rpm for 30 min, the supernatant was evaluated for the release of azo dye at 595 nm using spectrophotometer (Thermo Fisher Scientific, USA). Enzyme control (1.0 ml buffer + keratin azure suspension) was also prepared along with enzyme sample and incubated for 30 min at 50 °C, followed by addition of 2.0 ml TCA + 1.0 ml enzyme solution. One unit (U/ml) of keratinolytic activity was defined as an increase of corrected absorbance (0.01) at 595 nm, in relation to control using the formula:

$$ U=4\times n\times A595/\left(0.01\times 10\right) $$

where *n* is the dilution rate, 4 is the final reaction volume (ml), and 10 is the incubation time (min). Observations were obtained in triplicate for each sample.

### Selection of best conditions for keratinase activity

#### Production medium

The selection screening was done using the basal modified medium and following previously described methods [20]. Fermentation was carried out at 37 °C for 72 h with 150 rpm. The pH and volume of the medium were maintained at 7.5 and 50 ml, respectively. Three replicates were used for each experiment.

#### Effect of chicken feather concentrations

Various concentrations of feather meal were used in production media to identify the best concentration for maximum production of enzyme. The concentrations of feather meal were set to 0.25%, 0.5%, 1%, 1.5%, and 2% (w/v).

#### Effect of organic nitrogen sources

In the screening of various organic nitrogen sources, yeast extract (0.02%) in the basal media was replaced with tryptone, peptone, beef extract, and gelatin at the same concentration, individually.

#### Effect of inorganic nitrogen sources

In the screening of various inorganic nitrogen sources, NH_4_Cl (0.1%) in the basal media was replaced with NH_4_NO_3_, KNO_3_, NH_4_H_2_PO_4_, NH_4_SO_4_, and NaNO_3_ at the same concentration, individually.

#### Effect of carbon supplements

Additional 1% (w/v) carbon supplements were used in the fermentation process to find out the favorable carbon supplement. The supplements were starch, glucose, fructose, carboxymethyl cellulose (CMC), lactose, and sucrose.

#### Culture conditions

The culture conditions and replicates were maintained as described earlier in optimization for production medium [20]. Three replicates were used for each experiment.

#### Effect of incubation temperature and period

The effect of temperature on enzyme production was carried out by fermentation at different incubation temperatures from 25 to 45 °C (25, 30, 35, 40, and 45 °C). The effect of incubation period on the production of keratinase was investigated by fermenting the medium from 24 to 96 h at 37 °C (24, 48, 72, and 96 h). The sample was collected in every 24 h to observe the changes in keratinase production. The culture conditions were maintained as described earlier in optimization for production medium.

#### Effect of culture volume

The effects of different volumes of inoculum was investigated using 2%, 3%, 4%, 5%, 6%, and 7% inoculum in fermentation for the production of keratinase. The culture conditions were maintained as described earlier in optimization for production medium.

### Dehairing of goat skin

Goat skin was collected from a local slaughter house followed by washing with deionized distill water. The skin was treated with salt to protect the skin from rotting and dried in the presence of sunlight. Skin pieces (1 in. × 1 in.) were incubated in 20 ml crude keratinase at 37 °C for 72 h. Besides, a control dehairing was performed with physiological phosphate buffered saline (PBS) at the same conditions.

### Statistical analysis

One-way ANOVA with Tukey’s post hoc test was used to analyze all numerical data for this study. R studio was used for data analysis and plotting. In all cases, a *P* value of less than 0.05 was considered statistically significant.

## Results

### Isolation of bacteria

**Fig. 1:**
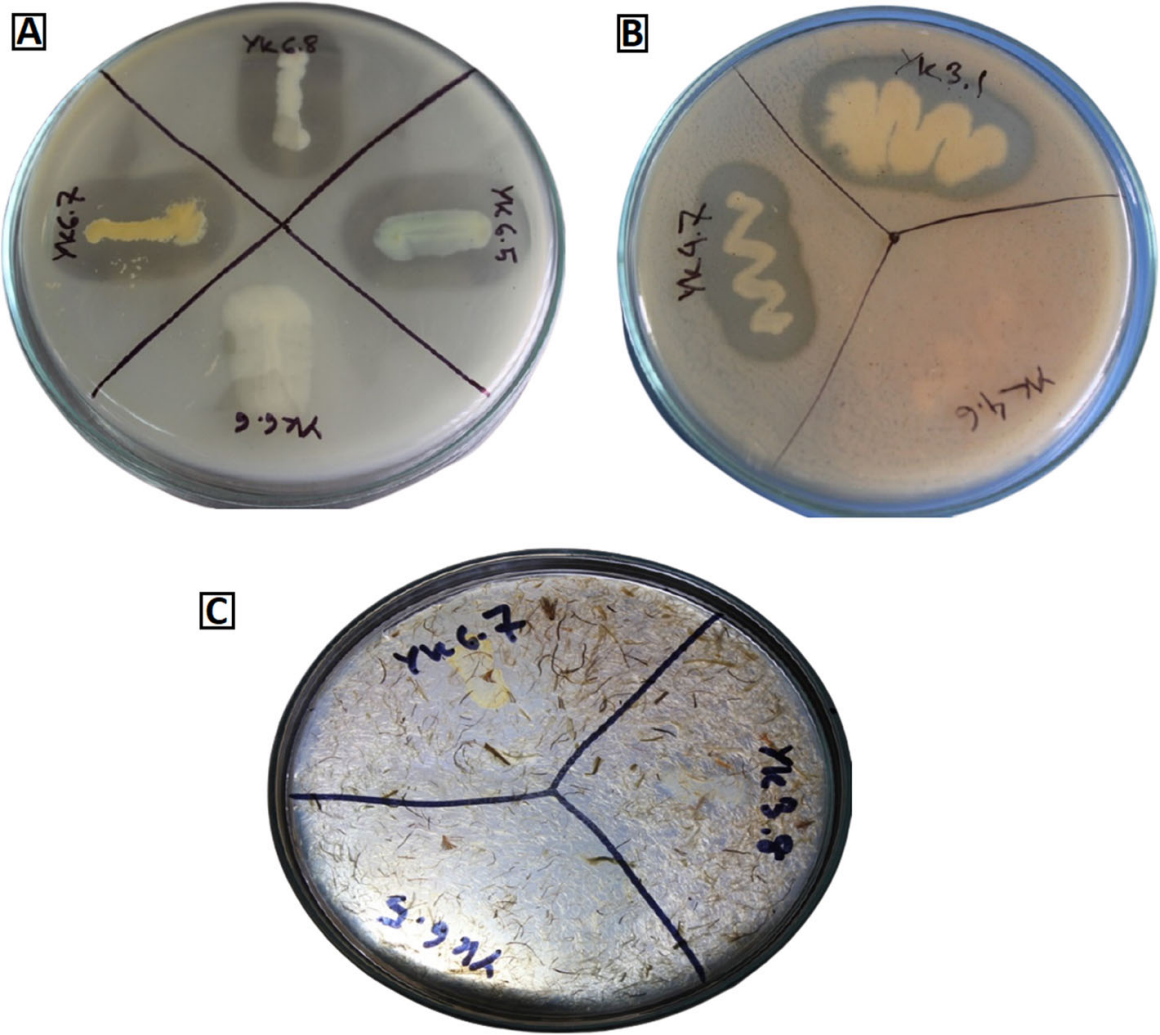
Keratinolytic activity of present study isolates. **a** Zone of clearance on skim milk agar. **b** Zone of clearance on casein agar. **c** Zone of clearance on feather agar

Based on biochemical and morphological characteristics (Table S1), a total of 50 isolates were screened for enzyme production assay. Out of 50, 16 were identified as keratinolytic bacteria based on the zone of clearance on skim milk (Fig. 1a), casein agar (Fig. 1b), and feather meal agar (Fig. 1c). Based on the enzyme activity on these media, eight potential isolates, namely, YK1.7, YK3.6, YK4.4, YK4.7, YK5.5, YK6.1, YK6.5, and YK6.7 were selected for further assays.

#### Selection of potent kertinolytic bacteria

**Fig. 2:**
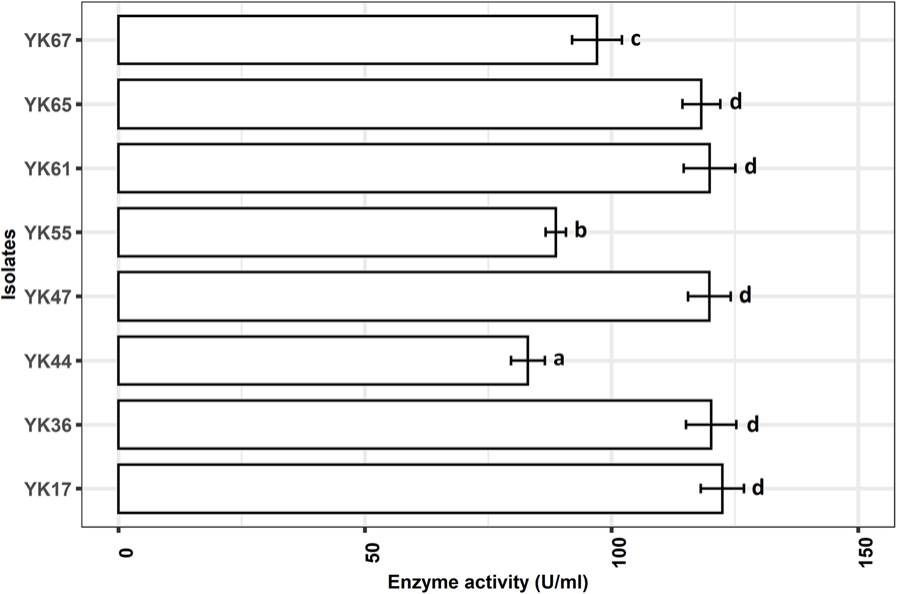
Production of keratinase (U/ml) by the present study isolates. Bar with different superscript indicates significantly different at *α* level of 0.05

Out of eight bacteria, four (YK17, YK36, YK47, and YK61) showed promising results in shake flask fermentation. The enzyme production from these bacteria was significantly (*P* < 0.05) higher than the other four bacteria (Fig. 2). However, isolate YK17 had the highest (*P* > 0.05) enzyme production in the shake flask compared to YK36. These two isolates were characterized further using 16S rRNA sequencing, and the highest enzyme producer YK17 was selected for the optimization.

#### Molecular characterization of bacteria

The phylogenetic analysis of 16S rRNA data classified the two most potent bacterial isolates as *Pseudomonas aeruginosa*. The nucleotide BLAST showed > 99.7% similarity to the sequence of respective species from the NCBI database. Phylogenetic tree revealed a close clustering of study isolates with some other keratinolytic strains from previous studies. Outgroup two strains of *Bacillus* were distantly clustered from the study isolates by forming a separate branch in the tree (Fig. S1).

#### Selection of best media conditions for the maximum production of enzyme

**Fig. 3:**
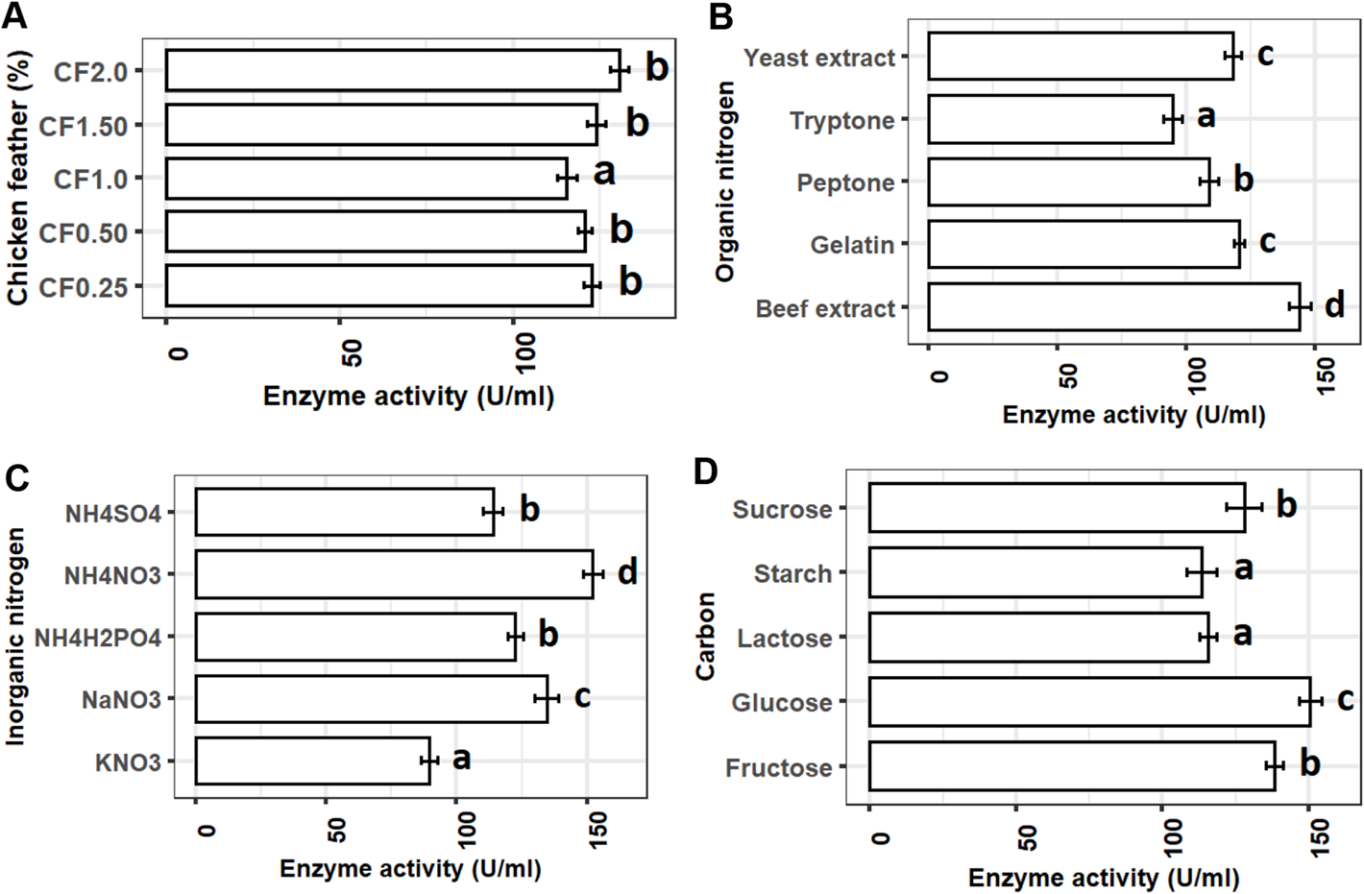
Effects of different media compositions on production of keratinase by the *P. aeruginosa* YK17 isolate. **a** Chicken feather (%). **b** Organic nitrogen sources. **c** Inorganic nitrogen sources. **d** Carbon sources. Bar with different superscripts indicates significantly different at *α* level of 0.05

Out of five different concentrations (0.25%, 0.5%, 1.0%, 1.5%, and 2%), the enzyme production was significantly lower only for 1% chicken feather supplements in the medium. Although the enzyme production was found higher with augmented chicken feather (1.5% and 2%) in the medium, the increased concentrations revealed to have no effects on production (Fig. 3a). Nitrogen sources in the medium have shown significant influence on enzyme production. Beef extract (144.9 U/ml) and ammonium nitrate (152.2 U/ml) have shown to be the most promising organic and inorganic sources of nitrogen for keratinase production using *P*. *aeruginosa* YK17. The enzyme production with these two supplements was significantly (*P* < 0.05) higher than other organic and inorganic nitrogen sources (Fig. 3b, c). Glucose was found as the most preferable carbon source for the optimum production of keratinase using *P*. *aeruginosa* YK17. The enzyme production was significantly higher (150.2 U/ml) with glucose compared to the other four carbon sources (Fig. 3d).

#### Selection of best culture conditions for the maximum production of enzyme

**Fig. 4:**
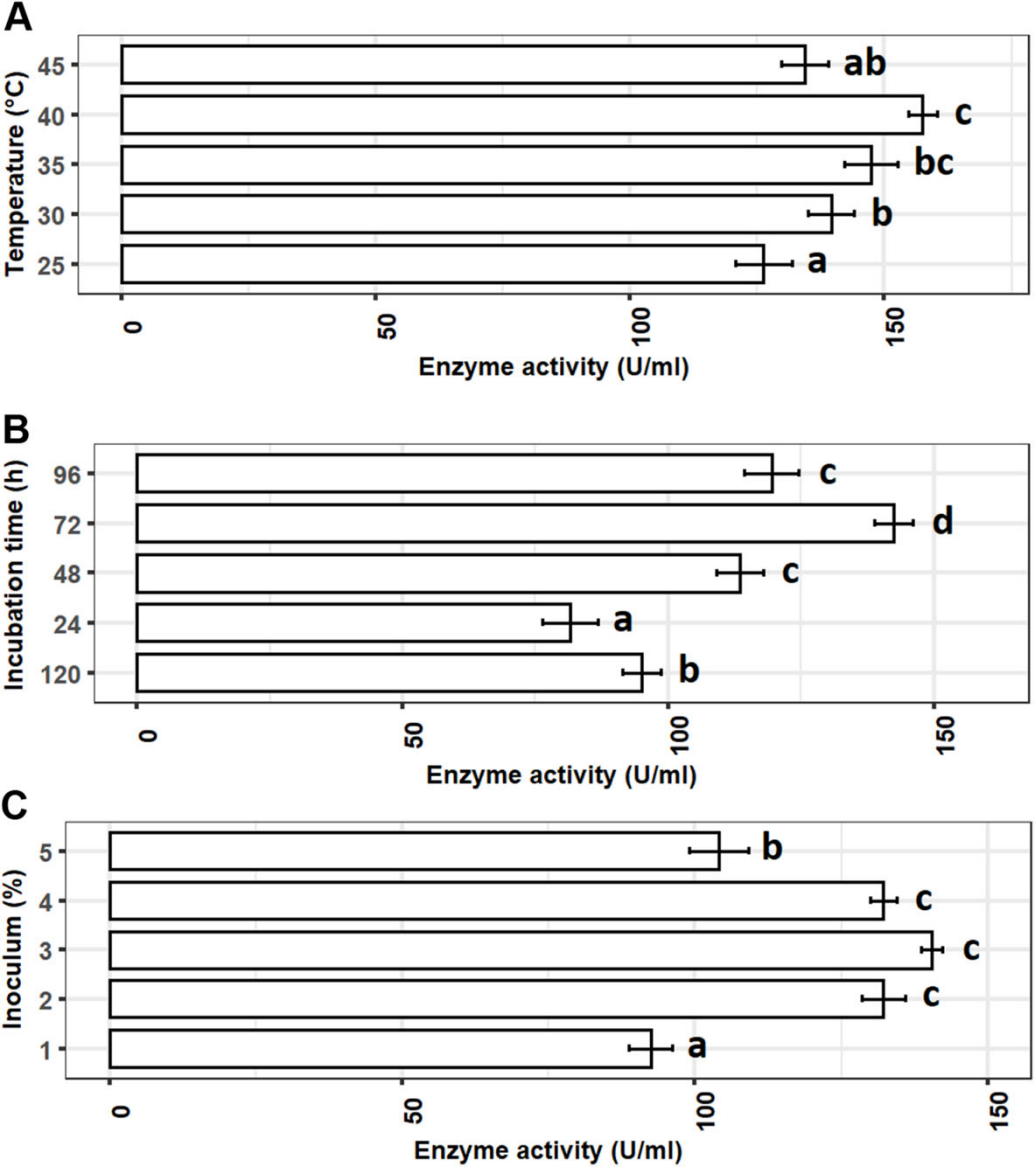
Effects of different culture conditions on production of keratinase by the *P. aeruginosa* YK17 isolate. **a** Temperature (°C). **b** Incubation time (h). **c** Inoculum (%). Bar with different superscripts indicates significantly different at *α* level of 0.05

The enzyme production was lowest (*P* < 0.05) with 25 °C incubation temperature compared to 30, 35, 40, and 45 °C. Temperature from 35 to 40 °C was found as the most suitable range for the optimum production of enzyme. After that, higher incubation temperature (45 °C) has shown negative impacts on enzyme production (Fig. 4a). A very similar trend was observed for incubation time where short incubation period (24 h) showed poor enzyme production. The enzyme production found increased with longer incubation where 72-h growth was found as the optimum time for the highest (*P* < 0.05) enzymatic activity. Afterwards, the enzyme activity decreased significantly with elongated incubation (96 and 120 h) (Fig. 4b). Finally, 2–4% inoculum (bacterial broth) was identified as the most suitable percentage range for optimum enzyme production, though the highest production was recorded at 3%. Both low (1%) and high (5%) percentages of inoculum significantly reduced the enzyme production by *P*. *aeruginosa* YK17 (Fig. 4c).

#### Dehairing of goat skin

**Fig. 5:**
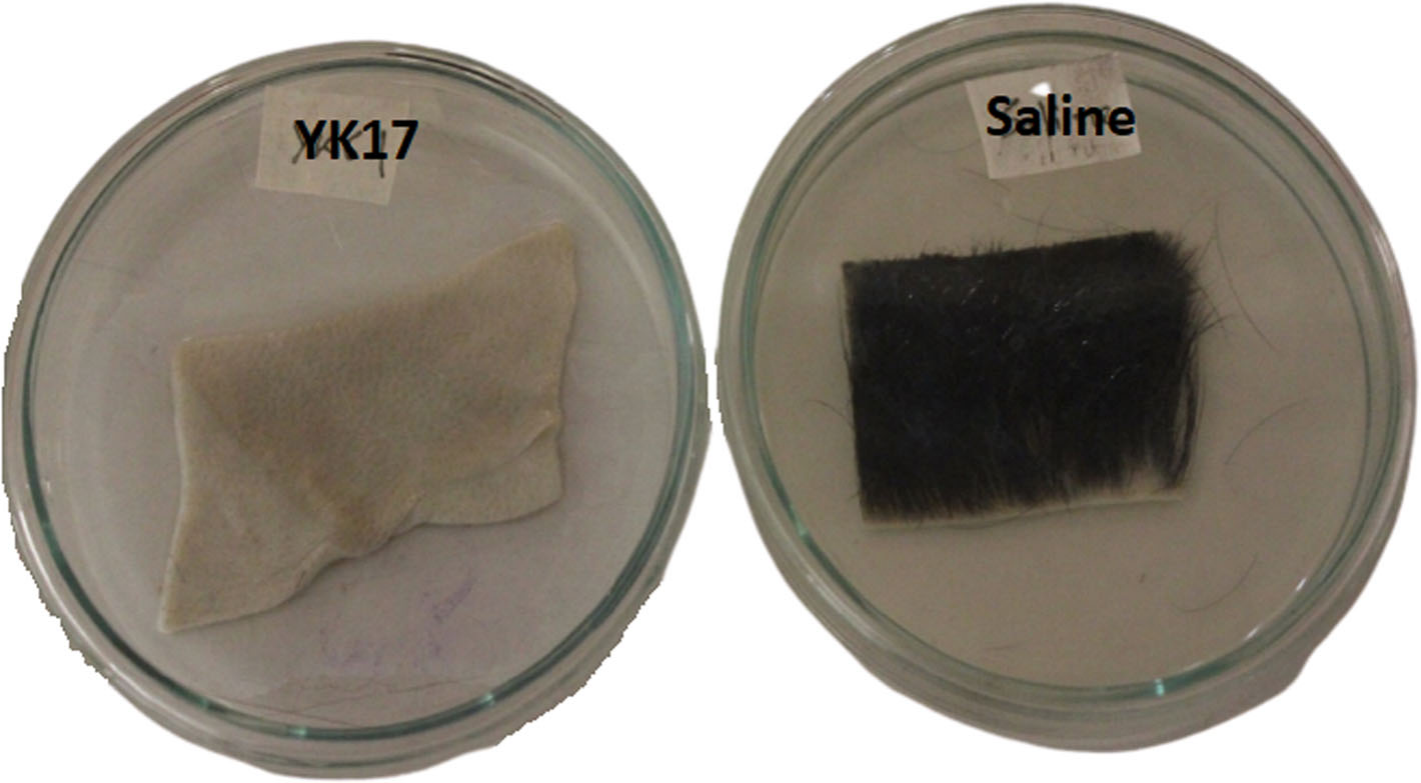
Dehairing of goat skin by *Pseudomonas aeruginosa* YK17 after 72 h

Goat skin was found completely dehaired after 72 h of incubation with 3% of 20 ml *P*. *aeruginosa* YK17 inoculum. At the same time, the control goat skin remained unaffected (haired) with PBS solution (Fig. 5).

## Discussion

Bacteria isolated from soil reported to have keratinolytic activity for industrial applications. Keratinase produced mostly by gram-positive bacteria, especially soil bacteria Actinomycetes and Bacilli [18, 32]. Majority of keratinases are belonging to serine proteases, a metalloprotease mostly synthesized by gram-negative bacteria [18]. Therefore, compared to gram-positive bacteria, gram-negative genera are more effective synthesizer of keratinase. In this context, some studies reported promising production of keratinase through gram-negative bacteria [33], especially *P*. *aeruginosa* [22]. However, none of the previous studies optimized the production parameters. In this study, alongside biochemical and molecular characterization, we have tested several combinations to select the best media compositions and culture conditions for keratinase production of gram-negative *P*. *aeruginosa* for the first time. Here, we initially screened 50 isolates from tannery soil for keratinolytic activity and found 30 gram-negative bacteria. The number of isolates with in vitro keratinase activity hence revealed that gram-negative bacteria are the most potential keratinase producer in Bangladeshi ternary soil.

In this study, the bacterial isolates were characterized following morphological and biochemical tests and 16S rRNA sequencing, a gold standard method for speciation of bacteria [34]. The phylogenetic analysis showed a close proximity and similarity index between two most potent enzyme-producing *P*. *aeruginosa* isolates YK17 and YK36. Other isolates in the same genera from previous studies formed separate branches specifying very dissimilar and distant strains of *P*. *aeruginosa*. A whole genome sequencing is therefore recommended to identify the functionally different genes and associated metabolic pathways.

The highest keratinase production of 122.2 U/ml from *P*. *aeruginosa* YK17 recorded for this study reached over 150 U/ml with suitable inorganic and carbon sources. This result is very promising and significant compared to a previous study that recorded almost half of keratinase production (80 U/ml) from *P*. *aeruginosa* C11 [22]. Three factors including highly polluted and diversified tannery soil in Bangladesh, sequence divergence, and high optimum temperature (40 °C) for maximum production of enzyme can be attributed to the higher production of enzyme by the present study isolates. Nevertheless, considering phylogenetic diversity and enzyme production, the present study isolates YK17 and YK36 are distinctly different from *P*. *aeruginosa* of other studies.

One of the most significant parts of this study was optimizing production parameters for the highest enzyme production for *P*. *aeruginosa*. High chicken feather (2%) in media is positively correlated to production of keratinase by *Bacillus subtilis* [35]. However, still now not much of studies have conducted on chicken feather optimization for gram-positive bacteria. Beef extract and ammonium nitrate as an organic and inorganic nitrogen showed the most suitable sources for maximum enzyme production. The results of ammonium nitrate are consistent with a previous study [20] where five different inorganic sources were used for the optimization of enzyme production by *Arthrobacter* sp. NFH5. However, the total production was 7 times higher in the present study with *P*. *aeruginosa* YK17. On the other hand, organic sources used in previous studies [20, 36] showed very much dissimilar results where authors reported yeast extract as the most suitable sources and we the found highest production with beef extract. Both of these studies tested suitable organic sources for the gram-positive bacteria which could be the reason of differences in organic nitrogen utilization and hence warrants further investigations. Carbon sources are one of the vital elements for bacterial growth, and *P*. *aeruginosa* YK17 in this study utilized glucose much efficiently than others. This results was very much different compared to previous studies with *Bacillus* sp. [36], *Amycolatopsis* sp. [37], and *Streptomyces* sp. [38] where authors reported starch, corn flour, and galactose as optimum carbon sources, and our study found glucose for *P*. *aeruginosa* YK17. Therefore, the most suitable carbon sources for the maximum production of enzyme by bacteria vary from one species to another.

In this study, we further optimized growth conditions for *P*. *aeruginosa* YK17, a mesophilic bacteria that can grow in a wide range of temperature between 20 and 45 °C, however reported to grow best between 37 and 40 °C. The optimum temperature for the highest enzyme production was found at 40 °C in the present study, and the results was consistent with previous studies of keratinase production from *Arthrobacter* sp. NFH5, *B. subtilis*, and *B. pumilis* [20, 39]. However, the temperature range used in present study only covered 35 and 40 °C not 37 °C, and therefore, it is not conclusive to state that 40 °C is the optimum temperature for maximum enzyme production. Considering this point and previous reports on higher optimum temperature of *P*. *aeruginosa*, further research is required optimize the temperature between 35 and 45 °C. The highest production was achieved after 72-h incubation. Initially, low enzymatic activity after 24-h incubation can be correlated with the lower concentration of bacteria in the shake flask. We found the highest enzymatic activity after 72-h incubation, and after that, negative competition due to log phase bacterial growth in the culture medium results in depletion of nutrients and hence lowered enzymatic activity. Finally, 2–4% inoculum volume was found as the most suitable range for the maximum enzymatic activity. Compared to *Arthrobacter* sp. NFH5 [20] that favored 24-h incubation and 5% inoculum, *P*. *aeruginosa* YK17 required low inoculum volume but higher incubation time for maximum keratinase production. Growth conditions and generation time of two different species possibly associated with the differences in inoculum volume and incubation time for the log phase growth.

## Conclusion

In present study, the results of keratinolytic protease production by the *P*. *aeruginosa* YK17 in this study was promising considering some other previous studies and dehairing experiment on goat skin. Thus, this eco-friendly enzymatic approach of dehairing could reduce the dependency on toxic chemical (sulfides, limes, and amides) used in leather industries and thereby improve public health outcomes. However, further research is required on optimization of pH, phosphate, substrates, and sample volume as well as sequencing of long reads using PacBio or Oxford Nano pore to develop an efficient *P*. *aeruginosa* YK17 strain for industrial applications.

## Supplementary Information

**Additional file 1: Table S1**. Morphological and biochemical characteristics of isolates. **Fig. S1**. Phylogenetic tree showing the relationship of present study *P. aeruginosa* isolates. GenBank accession number are given in the parentheses. The evolutionary distances were computed with Kimura 2-parameter model and tree was constructed using neighbor-joining method in MEGA 7.0

## Data Availability

All the data are available in the main manuscript and supplementary file.

## Abbreviations

DNA: Deoxy ribonucleic acids
PacBio: Pacific Biosciences
PCR: Polymerase chain reaction
