# Supplementary material for: Keratinolytic protease from Pseudomonas aeruginosa for leather skin processing
Source: J Genet Eng Biotechnol. 2021 Apr 6;19:53. doi: 10.1186/s43141-021-00149-8 (PMC8024431; doi:10.1186/s43141-021-00149-8)
Supplement: Supplementary file 1 — Additional file 1: Table S1. Morphological and biochemical characteristics of isolates. Fig. S1. Phylogenetic tree showing the relationship of present study P. aeruginosa isolates. GenBank accession number are given in the parentheses. The evolutionary distances were computed with Kimura 2-parameter model and tree was constructed using neighbor-joining method in MEGA 7.0 [file 43141_2021_149_MOESM1_ESM.docx]

**Table S1.** Morphological and biochemical characteristics of isolates

| SL | Isolate | Gram's test | Grams's staining | Shape | Catalase | Oxidase | Indole | Methyl red | Voges-Proskauer | Citrate | Salt tolerance | Nitrate reduction | Starch hydrolysis | Gelatin hydrolysis | MacConkey Agar |
| --- | --- | --- | --- | --- | --- | --- | --- | --- | --- | --- | --- | --- | --- | --- | --- |
|  | YK10 | + | + | Rod | + | + | - | - | + | + | + | + | - | + | + |
|  | YK11 | - | - | Rod | - | + | + | - | + | + | + | - | + | + | + |
|  | YK12 | + | + | Rod | + | + | - | - | + | + | + | + | - | + | + |
|  | YK13 | - | - | Rod | - | + | + | - | + | + | + | - | - | + | + |
|  | YK14 | + | + | Rod | + | + | - | - | + | + | + | + | - | + | + |
|  | YK15 | - | - | Rod | - | + | + | - | + | + | + | - | - | + | + |
|  | YK16 | + | + | Rod | + | + | - | - | + | + | + | + | - | + | + |
|  | YK17 | - | - | Rod | - | + | + | - | + | + | + | - | - | + | + |
|  | YK18 | - | - | Rod | - | + | + | - | + | + | + | - | - | + | + |
|  | YK19 | - | - | Rod | - | + | + | - | + | + | + | - | - | + | + |
|  | YK20 | + | + | Rod | + | + | - | - | + | + | + | + | - | + | + |
|  | YK30 | + | + | Rod | + | + | - | - | + | + | + | + | - | + | + |
|  | YK31 | - | - | Rod | - | + | + | - | + | + | + | - | - | + | + |
|  | YK32 | + | + | Rod | + | + | - | - | + | + | + | + | - | + | + |
|  | YK33 | - | - | Rod | - | + | + | - | + | + | + | - | - | + | + |
|  | YK34 | - | - | Rod | - | + | + | - | + | + | + | - | - | + | + |
|  | YK35 | - | - | Rod | - | + | + | - | + | + | + | - | - | + | + |
|  | YK36 | + | + | Rod | + | + | - | - | + | + | + | + | - | + | + |
|  | YK37 |  |  | Rod |  | + | + | - | + | + | + | - | - | + | + |
|  | YK38 | - | - | Rod | - | + | + | - | + | + | + | - | - | + | + |
|  | YK39 | - | - | Rod | - | + | + | - | + | + | + | - | - | + | + |
|  | YK40 | + | + | Cocci | - | + | - | - | - | + | + | + | - | + | + |
|  | YK41 | - | - | Rod | - | + | + | - | + | + | + | - | - | + | + |
|  | YK42 | + | + | Rod | + | + | - | - | + | + | + | - | - | + | + |
|  | YK43 | - | - | Rod | - | + | + | - | + | + | + | - | - | + | + |
|  | YK44 | + | + | Rod | + | + | - | - | + | + | + | + | - | + | + |
|  | YK45 | - | - | Rod | - | + | + | - | - | + | + | - | - | + | + |
|  | YK46 | - | - | Rod | - | + | + | - | + | + | + | - | - | + | + |
|  | YK47 | + | + | Rod | + | + | - | - | + | + | + | + | - | + | + |
|  | YK48 | - | - | Rod | - | + | + | - | + | + | + | - | - | + | + |
|  | YK49 | - | - | Rod | - | + | + | - | + | + | + | - | - | + | + |
|  | YK50 | - | - | Rod | - | + | + | - | + | + | + | - | - | + | + |
|  | YK51 | - | - | Rod | - | + | + | - | + | + | + | - | + | + | + |
|  | YK52 | + | + | Rod | + | + | - | - | + | + | + | + | - | + | + |
|  | YK53 | - | - | Rod | - | + | + | - | + | - | + | - | - | + | + |
|  | YK54 | - | - | Rod | - | + | + | - | + | + | + | - | - | + | + |
|  | YK55 | + | + | Rod | + | + | - | - | + | + | + | + | - | + | + |
|  | YK56 | + | + | Rod | + | + | - | - | + | + | + | + | - | + | + |
|  | YK57 | - | - | Rod | - | + | + | - | + | + | + | - | - | + | + |
|  | YK58 | + | + | Rod | + | + | - | - | + | + | + | + | - | + | + |
|  | YK59 | + | + | Rod | + | + | - | - | + | + | + | + | - | + | + |
|  | YK60 | - | - | Rod | - | + | + | - | + | + | + | - | - | + | + |
|  | YK61 | - | - | Rod | - | + | + | - | + | + | + | - | + | + | + |
|  | YK62 | + | + | Rod | + | + | - | - | + | + | + | + | - | + | + |
|  | YK63 | + | + | Rod | + | + | - | - | + | + | + | + | - | + | + |
|  | YK64 | + | + | Rod | + | + | - | - | + | + | + | + | - | + | + |
|  | YK65 | - | - | Rod | - | + | + | - | + | + | + | - | - | + | + |
|  | YK66 | - | - | Rod | - | + | + | - | + | + | + | - | + | + | + |
|  | YK67 | - | - | Rod | - | + | + | - | + | + | + | - | - | + | + |
|  | YK68 | - | - | Rod | - | + | + | - | + | + | + | - | - | + | + |





**Figure S1**. Phylogenetic tree showing the relationship of present study *P. aeruginosa* isolates. GenBank accession number are given in the parentheses. The evolutionary distances were computed with Kimura 2-parameter model and tree was constructed using neighbor-joining method in MEGA 7.0.
